# Rare Alleles and Signatures of Selection on the Immunodominant Domains of Pfs230 and Pfs48/45 in Malaria Parasites From Western Kenya

**DOI:** 10.3389/fgene.2022.867906

**Published:** 2022-05-17

**Authors:** Kevin O. Ochwedo, Fredrick O. Ariri, Wilfred O. Otambo, Edwin O. Magomere, Isaiah Debrah, Shirley A. Onyango, Pauline W. Orondo, Harrysone E. Atieli, Sidney O. Ogolla, Antony C. A. Otieno, Wolfgang R. Mukabana, Andrew K. Githeko, Ming-Chieh Lee, Guiyun Yan, Daibin Zhong, James W. Kazura

**Affiliations:** ^1^ Department of Biology, Faculty of Science and Technology, University of Nairobi, Nairobi, Kenya; ^2^ Sub-Saharan Africa International Centre for Excellence in Malaria Research, Homa Bay, Kenya; ^3^ Department of Zoology, School of Physical and Biological Sciences, Maseno University, Kisumu, Kenya; ^4^ Department of Biochemistry, Egerton University, Nakuru, Kenya; ^5^ West Africa Centre for Cell Biology of Infectious Pathogen, Department of Biochemistry, Cell and Molecular Biology, University of Ghana, Accra, Ghana; ^6^ School of Zoological Sciences, Kenyatta University, Nairobi, Kenya; ^7^ Centre for Global Health Research, Kenya Medical Research Institute, Kisumu, Kenya; ^8^ Program in Public Health, College of Health Sciences, University of California, Irvine, Irvine, CA, United States; ^9^ Centre for Global Health and Diseases, Case Western Reserve University, Cleveland, OH, United States

**Keywords:** Pfs230, Pfs48/45, transmission blocking vaccines, genetic diversity, evolutionary forces

## Abstract

**Background:** Malaria elimination and eradication efforts can be advanced by including transmission-blocking or reducing vaccines (TBVs) alongside existing interventions. Key transmission-blocking vaccine candidates, such as *Pfs230* domain one and *Pfs48/45* domain 3, should be genetically stable to avoid developing ineffective vaccines due to antigenic polymorphisms. We evaluated genetic polymorphism and temporal stability of *Pfs230* domain one and *Pfs48/45* domain three in *Plasmodium falciparum* parasites from western Kenya.

**Methods:** Dry blood spots on filter paper were collected from febrile malaria patients reporting to community health facilities in endemic areas of Homa Bay and Kisumu Counties and an epidemic-prone area of Kisii County in 2018 and 2019. *Plasmodium* speciation was performed using eluted DNA and real-time PCR. Amplification of the target domains of the two *Pfs* genes was performed on *P. falciparum* positive samples. We sequenced *Pfs230* domain one on 156 clinical isolates and *Pfs48/45* domain three on 118 clinical isolates to infer the levels of genetic variability, signatures of selection, genetic diversity indices and perform other evolutionary analyses.

**Results:** *Pfs230* domain one had low nucleotide diversity (*π* = 0.15 × 10^–2^) with slight variation per study site. Six polymorphic sites with nonsynonymous mutations and eight haplotypes were discovered. I539T was a novel variant, whereas G605S was nearing fixation*. Pfs48/45* domain three had a low *π* (0.063 × 10^–2^), high conservation index, and three segregating sites, resulting in nonsynonymous mutation and four haplotypes. Some loci of *Pfs230* D1 were in positive or negative linkage disequilibrium, had negative or positive selection signatures, and others (1813, 1955) and (1813, 1983) had a history of recombination. Mutated loci pairs in *Pfs48/45* domain three had negative linkage disequilibrium, and some had negative and positive Tajima’s *D* values with no history of recombination events.

**Conclusion:** The two transmission blocking vaccine candidates have low nucleotide diversity, a small number of zone-specific variants, high nucleotide conservation index, and high frequency of rare alleles. With the near fixation a polymorphic site and the proximity of mutated codons to antibody binding epitopes, it will be necessary to continue monitoring sequence modifications of these domains when designing TBVs that include Pfs230 and Pfs48/45 antigens.

## Introduction

Genetic polymorphism of *Plasmodium falciparum* antigens has hampered efforts to develop an effective vaccine that is protective against pre-erythrocytic and asexual blood-stage parasites (Genton et al., 2002; Takala et al., 2007; Ogutu et al., 2009; Bergmann-Leitner et al., 2012; Neafsey et al., 2015; Ouattara et al., 2015). Recent efforts, however, have been made to develop vaccines that reduce and block *Plasmodium falciparum* transmission at the community level. Two of the existing transmission-blocking vaccine (TBV) candidates, *P. falciparum* surface protein 230 (Pfs230) (Sabeti et al., 2007; Lee et al., 2019, 2020; Singh et al., 2019, 2020; Tachibana et al., 2019; Huang et al., 2020; Healy et al., 2021) and *P. falciparum* surface protein 48/45 (Pfs48/45) (Singh et al., 2019, 2021; Lee et al., 2020) have been shown to elicit antibody responses in mice and people that block *P. falciparum* gametocyte fertilization in the mid-gut of the *Anopheles* vector.

Pfs230 is a cysteine-rich 230 kDa protein expressed by both male and female gametocytes (Rener et al., 1983; MacDonald et al., 2016). The antigen is thought to play a role in gamete fusion in the mosquito blood meal after forming a complex with another cysteine-rich protein, Pfs48/45 (Eksi et al., 2006). In comparison to antibodies elicited by immunization with other Pfs230 domains, Domain 1 (D1) has been shown to elicit transmission-blocking monoclonal antibodies with strong inhibitory activity against oocyst development in standard membrane feeding assays (Lee et al., 2019; Singh et al., 2019, 2020; Tachibana et al., 2019; Huang et al., 2020; Healy et al., 2021). Like Pfs230 D1, fusion with its counterpart Pfs48/45 D3 has good potential as a component of a TBV. The latter fused doublet antigen consists of three domains linked by disulphide bonds and contains 16 cysteine residues (Kocken et al., 1993; Lennartz et al., 2018). Unlike Pfs230, Pfs48/45 is anchored on the gamete surface membrane by glycophophatidylinositol (Kocken et al., 1993; Dijk et al., 2001; Gilson et al., 2006; Lennartz et al., 2018) and is essential for male gamete fertility. Domain 3 has been shown to elicit antibodies in the host (Graves et al., 1988; Roeffen et al., 1994; Dijk et al., 2001; Bousema et al., 2010; Jones et al., 2015; Acquah et al., 2017; Singh et al., 2019; Baptista et al., 2022). Pfs48/45 D3 is located at the C-terminus of the protein and contains binding sites for non-inhibitory and inhibitory human and mouse mAbs that reduce *P. falciparum* infection in mosquitoes (Vermeulen et al., 1986; Graves et al., 1988; Outchkourov et al., 2007; Chowdhury et al., 2009; Singh et al., 2017, 2019; Kundu et al., 2018; Lennartz et al., 2018; Lee et al., 2020).

Antigenic polymorphism of Pfs230 D1 and Pf48/45 D3 should be assessed in malaria endemic areas on a regular basis to support the successful development of these TBV candidates due to the fact that, if the targeted regions are genetically unstable, polymorphisms may cause critical codon changes within immunogenic epitopes, thereby reducing TBV efficacy. Several dimorphic sites on Pfs230 D1 and Pfs48/45 D3 have previously been identified (Kocken et al., 1995; Drakeley et al., 1996; Escalante et al., 1998; Conway et al., 2001; Jones et al., 2015; MacDonald et al., 2016; Kundu et al., 2018; Singh et al., 2020; Coelho et al., 2021); however, there is limited knowledge of the extent of genetic diversity, signatures of selection, and other evolutionary forces that may be shaping alleles in *P. falciparum* from different malaria transmission zones. We therefore performed an in-depth genetic analysis of *Pfs230* D1 and *Pfs48/45* D3 in parasites isolated from patients with uncomplicated falciparum malaria from three different areas in western Kenya.

## Materials and Methods

### Study Site and Sampling

**FIGURE 1:**
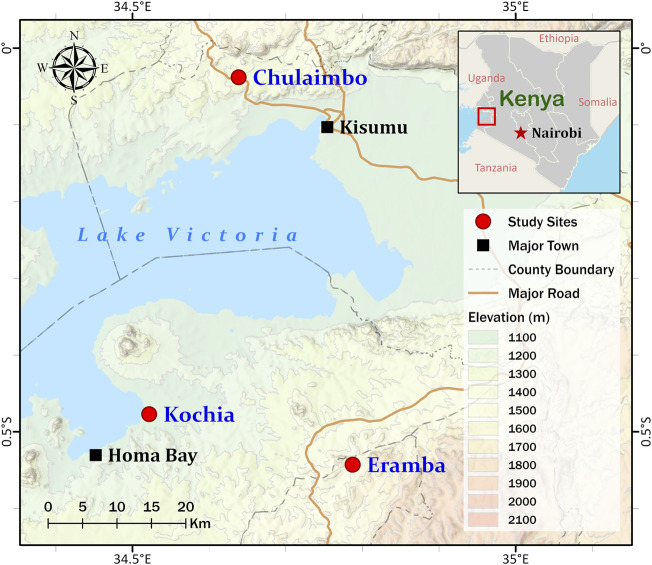
Map showing Homa Bay (Kochia), Kisumu (Chulaimbo) and Kisii (Eramba) study sites in western Kenya. The sampling points are represented by the red circles on the map. This figure was prepared with ESRI ArcGIS Pro 2.8 with field survey results and publicly available datasets. The map contains information from OpenStreetMap and OpenStreetMap 115 Foundation, which is made available under the Open Database License.

Dry blood spots (DBS) were collected on filter paper from febrile malaria patients at health clinics in Homa Bay County (Kochia), Kisumu County (Chulaimbo), and Kisii County (Eramba) in 2018 and 2019 (Figure 1). The study site in Homa Bay is characterized by perennial transmission. Vector control consists of universal distribution of long-lasting insecticidal bed nets with annual indoor residual spraying of insecticides. The study site in Kisumu County also has perennial transmission. Vector control consists of long-lasting insecticidal bed nets (LLINs) alone. The site in Kisii County is malaria epidemic-prone with low transmission and residents use LLINs (Kapesa et al., 2018). In brief, four drops of approximately 25 µL of blood from each patient were spotted on Whatman™ Blood Stain Cards (GE Healthcare WB100014) as previously described (Coombs and Fiscus, 2009). Each card was stored individually in silica gel-containing plastic bags before being transported to the joint International Centre of Excellence for Malaria Research (ICEMR) and Tom Mboya University College Laboratory in Homa Bay town for storage at -20°C. 150 of the 372 DBS collected came from Homa Bay; 120 and 102 came from Kisumu and Kisii, respectively.

### Amplification and Sequencing of *Pfs230* Domain one and *Pfs48/45* Domain three

Genomic DNA was extracted from filter paper using the modified Chelex resin (Chelex -100) method and stored at -20°C. As a positive control, DNA from the cultured laboratory strain NF54 was extracted and stored. *Plasmodium* species-specific real-time PCR targeting 18S ribosomal RNA gene was used to confirm *P. falciparum* positive DNA samples before amplification of specific target fragments of each gene (Ochwedo et al., 2021; Onyango et al., 2021). Primer sets were designed using Primer3 version 0.4.0 for *Pfs230* D1 and *Pfs48/45* D3 and in silico validation of each set was performed using the Sequence Manipulation Suite (Stothard, 2000). Among the 372 samples, 332 (89.3%) tested positive for *P. falciparum* DNA (n = 150, 120 and 62 from Homa Bay, Kisumu and Kisii, respectively) and were used to amplify *Pfs230* D1 and *Pfs48/45* D3 in a T100™ Thermal Cycler (Bio-Rad, Hercules, CA, United States ). Briefly, 3 µL of sample DNA was added to a mixture of 11.5 µL of DreamTaq Green PCR Master Mix (2X), 0.5 µL of *Pfs230* D1 forward (5′-TGG​TGA​AGC​TGT​CGA​AGA​TG-3′) and reverse primers (5′-GTG​TAC​CAC​AGG​GGG​AAG​AG-3′) targeting 514 base pairs and 7.5 µL of double-distilled water. The thermal profile was set as follows 95 °C for 3 min, 34 cycles (94 °C for 30 s, 58 °C for 30 s and 72 °C for 45 s) and final extension at 72 °C for 6 min. For *Pfs48/45* D3, similar reaction volume was prepared using forward (5′-TTT​TCA​AGA​AGG​AAA​AGA​AAA​AGC-3′) and reverse primers (5′-GCC​AAA​AAT​CCA​TAA​TAT​GCT​GA-3′) targeting 600bp. The PCR conditions were set as follows 95°C for 3 min, 34 cycles (94°C for 30 s, 55 °C for 30 s and extension at 72°C for 45 s) final extension at 72 °C for 6 min. All the amplicons were assessed by gel electrophoresis in 1.5% w/v agarose gel before sequencing. For *Pfs230* D1, 82, 39, and 35 samples from Homa Bay, Kisumu, and Kisii, respectively, were amplified. For *Pfs48/45* D3, 36, 44, and 38 samples from Homa Bay, Kisumu, and Kisii, respectively, were amplified. All the PCR amplicons, together with positive controls, were purified using Exonuclease I and Shrimp Alkaline Phosphatase (ExoSAP-IT) and bi-directionally sequenced using 3730 BigDye® Terminator v3.1 Sequencing Standard kit on ABI PRISM® 3700 DNA Analyzer (Applied Biosystems, Foster City, CA, United States).

### Data Analysis

All sequences were assembled using Geneious version 11.1.5 software, and multiple sequence alignment was performed using ClustalW. Polymorphic locus and codons were inferred after comparing each sequence to the respective sequence of positive control (NF54) as well as 3D7 (PF3D7_0209000 for *Pfs230* and PF3D7_1346700 for *Pfs48/45*). DnaSP Version 6.12.03 (Rozas et al., 2017) and Arlequin version 3.5.2 (Excoffier and Lischer, 2010) were used to compute genetic diversity indices such as nucleotide diversity (*π*), haplotype diversity (Hd), number of haplotypes (h), number of segregating sites (S) and mean number of pairwise difference (k). Population Analysis with Reticulate Trees (Popart) version 1.7 software (Clement et al., 2000) was used to infer haplotype networks. Neutrality tests; Tajima’s *D*, Fu and Li’s D, Fu and Li’s F and Fu’s Fs statistics and test for the presence of Recombination events (Rm) and linkage disequilibrium (LD) were computed in DnaSP Version 6.12.03 and Arlequin version 3.5.2. Generated Tajima’s *D* values were plotted using GraphPad version 8.3.0. Both antigen structural delineation was done using Protein Homology/analogY Recognition Engine (PHYRE2) version 2.0 and generated models visualized and edited in UCSF Chimera version 1.15 (Pettersen et al., 2004).

## Results

### Analysis of Mutations Detected in *Pfs230* D1 and *Pfs48/45* D3

Six loci (1,616, 1813, 1955, 1964, 1967, and 1983) in *Pfs230* D1 were found to be polymorphic, resulting in nonsynonymous mutations (Table 1). The mutations were skewed toward transversion, with a transversion to transition ratio (Tv: Ts) greater than 0.5. Two polymorphic sites were singletons (1964 and 1967), whereas four dimorphic sites (1,616, 1813, 1955, and 1983) were parsimony informative. These polymorphisms resulted in I539T, G605S, T652R, E655V, T656N, and K661N codon changes. Nonsynonymous alterations T652R and K661N were on separate beta (*β*) pleated sheets connected by a loop containing mutated codons E655V and T656N (Supplementary Figure S1). G605S was also on the loop connecting two different *ß* pleated sheets. In general, western Kenya parasites had a high allelic frequency of G605S (98.08%), followed by progressively lower frequencies of K661N, T652R, and I539T. E655R and T656N were each observed at a frequency of <1%. The prevalence of various alleles was almost similar across the various study sites. For example, as shown in Table 1, G605S was the most common codon change in the three study sites. Only two *P. falciparum* isolates from Kisumu and Homa Bay County lacked this mutation.

**TABLE 1:** Polymorphic sites on *Pfs230* domain one and *Pfs48/45* domain two and three from Homa Bay, Kisumu and Kisii region in western Kenya n: number of sequences harbouring mutations; *: reference (3D7and NF54) allele only; D: Domain; Nsyn: Non-synonymous mutation; Syn: Synonymous mutation; A: Adenine; C: Cytosine; T: Thymine; G: Guanine.

*Pfs230* (N = 156)								
Segregating Sites	Domains	Allelic Frequency			Substituted Bases	Type of substitution	Codon Change	Type of Mutation
		Homa Bay n (%)	Kisumu n (%)	Kisii n (%)				
1616	D1	-	2 (5.1)	-	*T/C	Transition	I539T	Nsyn
1813	D1	81 (98.8)	37 (94.9)	35 (100)	*G/A	Transition	G605S	Nsyn
1955	D1	2 (2.4)	1 (2.6)	2 (5.7)	*C/G	Transversion	T652R	Nsyn
1964	D1	-	-	1 (2.9)	*A/T	Transversion	E655V	Nsyn
1967	D1	-	1 (2.6)	-	*C/A	Transversion	T656N	Nsyn
1983	D1	35 (42.7)	19 (48.7)	16 (45.7)	*A/C	Transversion	K661N	Nsyn
Pfs48/45 (N = 118)								
753	D2	-	-	1 (2.6)	*T/C	Transition	Y251Y	Syn
757	D2	-	4 (9.1)	3 (7.9)	*A/G	Transition	K253E	Nsyn
762	D2	1 (2.8)	3 (6.8)	3 (7.9)	*C/G	Transversion	N254K	Nsyn
911	D3	1 (2.8)	-	-	*T/A	Transition	V304D	Nsyn
940	D3	3 (8.3)	3 (6.8)	9 (23.7)	*T/A	Transition	L314I	Nsyn
979	D3	-	-	1 (2.6)	*T/G	Transversion	C327G	Nsyn

In contrast to the six nonsynonomous mutated sites observed in *Pfs230* D1, *Pfs48/45* D3 had three segregating sites (Table 1). Singleton sites were found at loci 911 and 979 in parasites isolated from patients residing in Homa Bay County and Kisii County, respectively. A low frequency polymorphism at locus 940 was observed across parasite populations in all three counties, and was parsimony-informative. These transition bias mutations at loci 911, 940, and 979 resulted in nonsynonymous mutations V304D, L314I, and C327G, respectively. The variants were in the Pfs48/45 D3 antigen loop connecting different *ß* pleated sheets (Supplementary Figure S1). Codon change C327G in D3 was found in only one sequence in parasites isolated from a patient in Kisii County (Table 1). The ability of the designed primer set to cover *Pfs48/45* D3 also allowed for the discovery of a singleton site 753 (Y251Y) and parsimony-informative sites 757 and 762 (K253E and N254K) (Table 1; Supplementary Figure S1). Except for G605S, which was near dimorphic codons on Pfs48/45 domain 2, the superimposed structure revealed dimorphic codons of Pfs48/45 D3 antigen close to those of Pfs230 D1 (Supplementary Figure S2).

### Genetic Diversity of *Pfs230* and *Pfs48/45* Genes in Western Kenya

**FIGURE 2:**
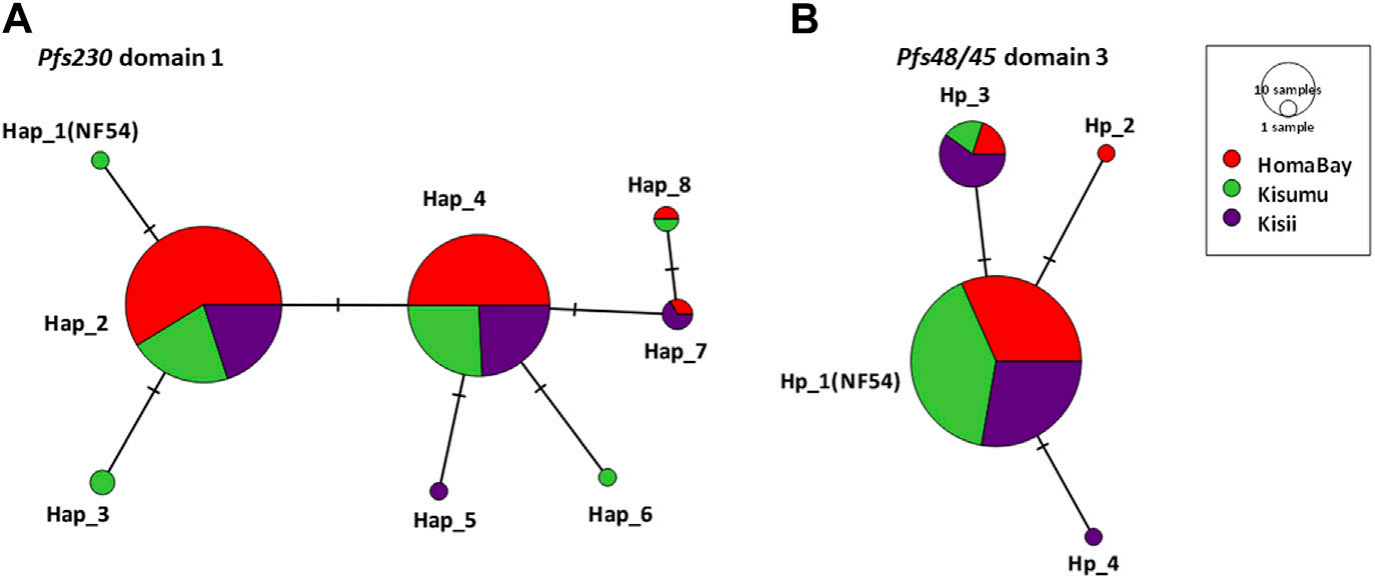
TCS-network analysis of the relationship of haplotypes based on the of *Pfs230* domain one and *Pfs48/45* domain three from parasites in malaria-endemic and epidemic-prone regions of western Kenya. **(A)**. represents haplotypes based on observed sequence variation in *Pfs230* D1 whereas **(B)** represents haplotypes in *Pfs48/45* D3. The network shows the distribution of haplotype within malaria-endemic Homa Bay (red) and Kisumu (Chulaimbo) (green) as well as epidemic Kisii highland (purple). The hatch marks represent the number of mutations resulting in a specific haplotype whereas the size of the circle equates to the frequency of the observed haplotypes. Haplotypes labelled Hap_1(NF54) for *Pfs230* D1 and Hp-1 (NF54) for *Pfs48/45* D3 lacked mutated locus and have 100% sequence identity to laboratory strain NF54 or 3D7. Nucleotide sequences of all haplotypes were submitted to GenBank (accession: MW624857- MW625101).

*Pfs230* D1 from the three sites had *π* of 0.15 × 10^–2^, k; 0.68 and haplotype diversity (Hd) of 0.57 (Table 2). The domain had a nucleotide conservation index of 98.7% with a total of eight haplotypes circulating in western Kenya (Figure 2). Kisumu had the highest *π* (0.18 × 10^–2^) followed by Kisii (0.15 × 10^–2^) and Homa Bay (0.12 × 10^–2^). The site also had the most haplotypes 6) and the highest Hd (0.63) when compared to Kisii and Homa Bay, which had four haplotypes each and Hd of 0.60 and 0.52, respectively (Figure 2). Haplotype 2 (Hap_2) with the mutated codon G605S was the most common in western Kenya and at each study site. This was followed by Hap_4 (mutated codons G605S and K661N), Hap_7 (G605S, T652R, and K661N), and Hap_8 (T652R and K661N) (Supplementary Table S1). The remaining haplotypes (Hap_1, Hap_3, Hap_6, and Hap_5) were observed at a lower frequency. Only one sequence (from Chulaimbo) in western Kenya lacked a mutated site and had 100% sequence identity to the laboratory strain PF3D7_0209000 or NF54 sequence used as a positive control (Figure 2).

**TABLE 2:** Summary of genetic diversity indices for *Pfs230* domain one and *Pfs48/45* domain three from parasites in western Kenya N: Sample size; C: Conservation index; S: Segregating sites; *π*: nucleotide diversity; Vars: Variants; Hd: Haplotype diversity.

*Pfs230* D1 Region	N	C (%)	S	π (×10^–2^)	h	Hd	Tajima’s *D*	Fu’s F_S_	FLD*	FLF*
Homa Bay	82	99.40	3	0.12	4	0.52	−0.11	−0.21	−0.54	−0.45
Kisumu	39	98.90	5	0.18	6	0.63	−0.80	−1.94	−0.73	−0.82
Kisii	35	99.40	3	0.15	4	0.60	−0.15	−0.39	−0.31	−0.29
W. Kenya	156	98.70	6	0.14	8	0.56	−0.82	−3.33	−0.94	−1.03
*Pfs48/45* D3										
Homa Bay	36	99.50	2	0.05	3	0.21	−1.09	−1.42	−0.80	−0.95
Kisumu	44	99.80	1	0.03	2	0.13	−0.60	−0.30	0.55	0.244
Kisii	38	99.50	2	0.10	3	0.41	−0.21	−0.12	−0.81	−0.74
W. Kenya	118	99.30	3	0.06	4	0.25	−0.94	−1.87	−2.06	−2.00

The *Pfs48/45* D3 from western Kenya had low *π* (0.06 × 10^–2^_)_ and Hd (0.25) (Table 2). The domain had a conservation index of 99.3%, with four haplotypes circulating in the study area (Figure 2). Kisii parasites had the highest *π* (0.10 × 10^–2^) followed by Homa Bay (0.05 × 10^–2^) and Kisumu (0.03 × 10^–2^) (Table 2). Kisii and Homa Bay study sites each had three haplotypes in circulation, while Kisumu had four (Figure 2). The majority of haplotypes lacked a mutation (Hp_1) or had 100% sequence identity to the laboratory strain PF3D7_1346700 or the NF54 sequence used as a positive control (85.6%). This was followed by Hp_3 (mutated codon L314I), which had an overall frequency of 12.7%, while the rest (Hp_2 and Hp_4) had a frequency <1% (Supplementary Table S2).

### Signatures of Selection, Linkage Disequilibrium and Recombination Events

**FIGURE 3:**
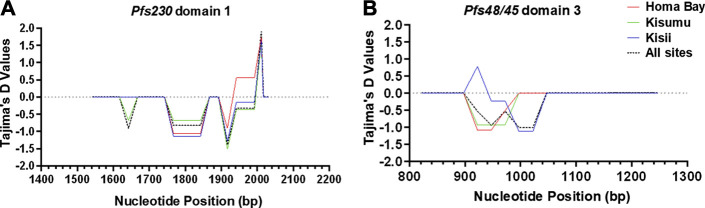
Sliding window plot of Tajima’s *D* values for *Pfs230* domain one and *Pfs48/45* domain three in western Kenya. The *X*-axis displays the nucleotide position (Window midpoint) whereas the Tajima’s *D* values are represented on the *Y*-axis. **(A)** is a representation of *Pfs230* domain one whereas **(B)** represents of *Pfs48/45* domain 3. The blue curve represents computed Tajima’s *D* plot for sequences of *P. falciparum* circulating in Kisii, the red colour is for Homa Bay, the green colour is for Kisumu (Chulaimbo) whereas the black dotted colour represents the population from the three study sites. The middle horizontal dotted line (intersecting the *Y*-axis at 0.0) represents a standard neutral model where the Tajima’s *D* value is equal to zero. Positive deviation from the grey dotted line signifies balancing selection whereas negative deviation represents purifying selection.

*Pfs230* D1 from *P. falciparum* isolates deviated from a standard neutral model. Tajima’s *D* (-0.8), FLD* (-0.9) and FLF* (-1.0) tests were all negative and non-significant (*p* > 0.05) (Table 2). However, Fu’s F_S_ result (-3.3), was significant (*p* = 0.023). Tajima’s *D* test results were also non-significant (*p* > 0.05) in each site (Table 2). Despite the overall negative Tajima’s D results, there was a slight variation among individual mutated loci on *Pfs230* D1. Locus 1983 (codon change K661N) had a significant (*p* < 0.05) positive (1.9) Tajima’s *D* result, whereas the rest had negative results (Figure 3). Apart from deviating from a standard neutral model, some loci pairs (1813, 1955) had positive LD (D′) results with highly significant (*p* < 0.001) χ ^2^ values of 19.7 and 12.7 among Homa Bay and Kisumu sequences, respectively (Supplementary Table S1). Among Kisii sequences, loci pairs (1813 and 1983) had positive D′ results, despite the fact that the χ ^2^ values was non-significant and the *r* ^2^ value was low (0.03). Other loci with positive D′ results, low *r* ^2^ values and non-significant (*p* > 0.05) χ^2^ values in Homa Bay and Kisumu parasites include loci pairs (1813,1983) (1955,1983) and (1967,1983). Some loci pairs on Kisumu and Kisii sequences had negative D′ results (Supplementary Table S1 **)**. On *Pfs230* D1 from parasites in Homa Bay and Kisumu, recombination events were detected across loci pairs (1813, 1955) and (1813, 1983) (Supplementary Table S1).

All of the *Pfs48/45* D3 sequences had non-significant (*p* > 0.05) negative Tajima’s *D* -1.9, FLD*: 2.1 and FLF*: 2.0 results. The Fu’s F_S_ (-1.9) result was, however, significant (*p* = 0.096). Locus 940 (L314I) among Kisii sequences had a significant (*p* > 0.05) positive Tajima’s *D* (0.8) results (Figure 3). There was no evidence of Rm or positive D′ results at any of the dimorphic loci within *Pfs48/45* D3. However, some loci pairs from Homa Bay and Kisii had negative D′ results (Supplementary Table S2).

## Discussion

The Pfs230 D1 and Pfs48/45 D3 antigens are important candidate antigens in the development of an effective TBV. Despite having a high frequency of rare alleles in western Kenya, both targets had low nucleotide diversity. Two variants, each on Pfs230 D1 and Pfs48/45 D3, were novel and private to western Kenya. The study validated five previously described polymorphic sites on *Pfs230* D1 (Singh et al., 2020). In this study, G605S, one of the five mutated codons, was fixed in some study areas but not in others. *Pfs230* D1 had the most mutations, while the Pfs48/45 D3 was the most conserved. Mutated loci from both domains were either under purifying or balancing selections. Other genetic forces revealed to have shaped alleles on the two genes included inbreeding and genetic drift with recombination being discovered only *Pfs230* D1.

*Pfs230* D1 from western Kenya had low nucleotide diversity, with significant Fu’s F_S_ results indicating a high frequency of rare alleles. In addition to the previously reported 15 polymorphisms on *Pfs230* D1 from parasites in Asian (Bangladesh, Cambodia, Laos, Myanmar, Thailand, and Vietnam) and African (Democratic Republic of the Congo, Ghana, Guinea, Malawi, Mali, Nigeria, Senegal, and Gambia) countries (MacDonald et al., 2016; Singh et al., 2020), this study discovered one additional mutation (I539T). This novel variant was identified only at the Kisumu study site along with five other polymorphisms (G605S, T652R, E655V, T656N, and K661N). These findings validate five previously described polymorphisms reported by (Singh et al., 2020). We speculate that the relatively higher nucleotide diversity index and number of haplotypes in Kisumu compared to other sites in western Kenya is related to the region’s slightly higher malaria transmission and absence of IRS activities (Oduma et al., 2021; Ochwedo et al., 2022).

Missense mutation G605S was found in parasites at a slightly higher allelic frequency (AF = 0.98) than in other geographical regions, as described by (Singh et al., 2020) (AF = 0.94) (MacDonald et al., 2016), (AF = 0.11), and (Coelho et al., 2021) (AF = 0.91). With only two clinical isolates in western Kenya lacking this mutation, G605S is almost completely fixed. This indicates the presence of selection pressure from either host antibodies, vector immune response or genetic drift (decreased variation and increasing homozygosity), may be stronger on *P. falciparum* populations from Kisii (low parasite population size) or Homa Bay (endemic site with declining parasite population size) compared to Kisumu (Chulaimbo) (Hancock and Di Rienzo, 2008; Honnay, 2013; Oduma et al., 2021). In contrast, the second most common polymorphism, K661N, was found in Kisumu at a higher frequency than in Kisii and Homa Bay. This reversal in the observed G605S and K661N frequencies could be attributed to factors such as recombination events (Rm), which are known to interfere with linked loci and could be effective on linked dimorphic loci pair 1813 and 1983 (responsible for G605S and K661N mutations respectively), thus increasing diversity in Kisumu (Chulaimbo) (Mejia, 2012) as opposed to Kisii and Homa Bay parasites, which also lack Rm between the two sites. Since immunogenic epitope binding light chain of transmission-blocking 4F12 monoclonal antibodies (TB 4F12 mAb) is close to the dimorphic codon G605, selection pressure from host antibodies on the epitope may be affecting the surrounding codons (MacDonald et al., 2016; Singh et al., 2020). This codon is located within a disulphide loop (from 593 to 611) that is thought to be stabilizing the epitope binding of TB 4F12 mAb (Singh et al., 2020). With near-complete fixation, the mutation may be beneficial to parasites but have a negative effect on the epitope binding affinity of TB 4F12 mAb. This needs to be looked into further by immunoassays of haplotypes with this polymorphism. Polymorphism I539T was found near codons 542–592 that contain 3G2 and 5G3 mAb binding epitopes which were previously shown to have no detectable oocyst reduction activity (Singh et al., 2020). Other polymorphisms, T652R, K661N on different *ß* pleated sheets, and E655V, T656N on disulphide loops linking the two loops, were distally located from the epitope that binds TB 4F12 mAb (Singh et al., 2020). When the two fusion proteins were superimposed, these four polymorphic codons were closer to mutated codon V304D, L314I, and C327G on Pfs48/45 D3, supporting the hypothesis that antibodies could be sterically interfering with protein-protein interaction (Singh et al., 2020). *Plasmodium falciparum* may induce these mutations in response to antibody-induced pressure in order to circumvent the blockade of fusion between Pfs230 D1 and Pfs48/45 D3, resulting in an uninterrupted gametocyte fertilization process.

The novel missense polymorphism C327G on Pfs48/45 D3 has the potential to be very important because it can interfere with one of the six cysteine residue pairings (pairing between codon C298 and C327) on the 85RF45.1 mAb epitope (Kundu et al., 2018). Other polymorphisms (Y251Y, K253E, N254K in Pfs48/45 D2 and V304D, L314I in Pfs48/45 D3) have been observed in *P. falciparum* populations in other malaria endemic regions (Conway et al., 2001; Jones et al., 2015; Kundu et al., 2018). However, none of these polymorphisms had been previously reported by a study conducted in the Asembo Bay area of western Kenya (Escalante et al., 1998). Though not the focus of this study, polymorphisms on codon 254 is thought to influence the type of host antibody that binds at the epitope bearing this mutation on Pfs48/45 antigen (Kocken et al., 1995). The three polymorphic codons Y251Y, K253E, and N254K on Pfs48/45 D2, are close to the disulphide loop that stabilizes the epitope binding TB 4F12 mAb on Pfs230 D1, thus suggesting steric interference from the antibodies. *Pfs48/45* domain three is highly conserved, with low nucleotide and haplotype diversity when compared to *Pfs230* D1. The key polymorphism based on this domain was L314I, which has a higher allelic frequency in Kisii highlands than in Homa Bay and Kisumu. Despite the presence of a high frequency of rare alleles, the majority of parasites lacked polymorphic loci on *Pfs48/45* D3.

Inbreeding, recombination, and natural selection were identified as major drivers of the observed mutations in *Pfs230* D1 and *Pfs48/45* D3. The presence of linkage disequilibrium confirmed the history of selection pressure and inbreeding across various loci in *Pfs230* D1 and *Pfs48/45* D3 (Larrañaga et al., 2013). Some polymorphisms were considered intermediary because they had negative linkage disequilibrium (D′) values (Silvela et al., 1999). The negative D′ values also confirmed a history of random drift, which is decreasing the number of variants while increasing homozygosity that may play a role in the parasite’s loss of favourable mutations if it persists (Barton, 2010).

The presence of natural selection was confirmed by the Tajima’s D values. Overall negative Tajima’s *D* results revealed that purifying selection was affecting the majority of loci within *Pfs230* D1 and *Pfs48/45* D3, reducing genetic diversity (Cvijović et al., 2018). The aforementioned selection was, however, weak because the computed negative Tajima’s *D* values in both antigens were not significant. Individual Tajima’s *D* results for each codon revealed all other dimorphic codons to be under purifying selection, with the exception of K661N on Pfs230 D1 from all study sites and V304D on Pfs48/45 D3 from Kisii, which are under strong and weak balancing selection, respectively. The two mutated loci under balancing selection may play an important role within the Pfs230 D1 and Pfs48/45 D3 fusion proteins, which may explain why they are maintained in the *P. falciparum* population from western Kenya (Escalante et al., 1998). These findings support previous postulation (Jones et al., 2015) that selection pressure is acting on immunogenic domains of Pfs48/45.

The presence of weak purifying selection acting on dimorphic sites may impact not only host mAb binding and functional activity but also be affected by selective pressure in the mosquito vector (Lombardo and Christophides, 2016). This pressure could be exerted on individual antigens before or after complex formation. Findings in the present study support future investigations that examine functional antibody responses such as the ability of PfS230 and Pfs48/45 antibodies that activate human plasma complement and reduce mosquito infectivity in membrane feeding assays.

## Conclusion

The *Pfs230* D1 and *Pfs48/45* D3 in *P. falciparum* from western Kenya have low nucleotide diversity and a high conservation index with high frequency of rare alleles. Among the observed polymorphisms in *Pfs230* D1, G605S is nearly fixed in the population. Natural selection, inbreeding, and, to some extent, recombination are important driving forces in shaping these alleles in the two antigens. With the discovery of novel polymorphic sites, the two domains of the *Pfs230* and *Pfs48/45* from different malaria-prone regions, including areas where clinical trials have been conducted, should be monitored indefinitely. This will help track the genetic stability of the two TBV candidates.

## Data Availability

The datasets presented in this study can be found in online repositories. The names of the repository/repositories and accession number(s) can be found in the article/Supplementary Material.
