# Supplementary figures and images for: Rare Alleles and Signatures of Selection on the Immunodominant Domains of Pfs230 and Pfs48/45 in Malaria Parasites From Western Kenya
Source: Front Genet. 2022 May 17;13:867906. doi: 10.3389/fgene.2022.867906 (PMC9152164; doi:10.3389/fgene.2022.867906)

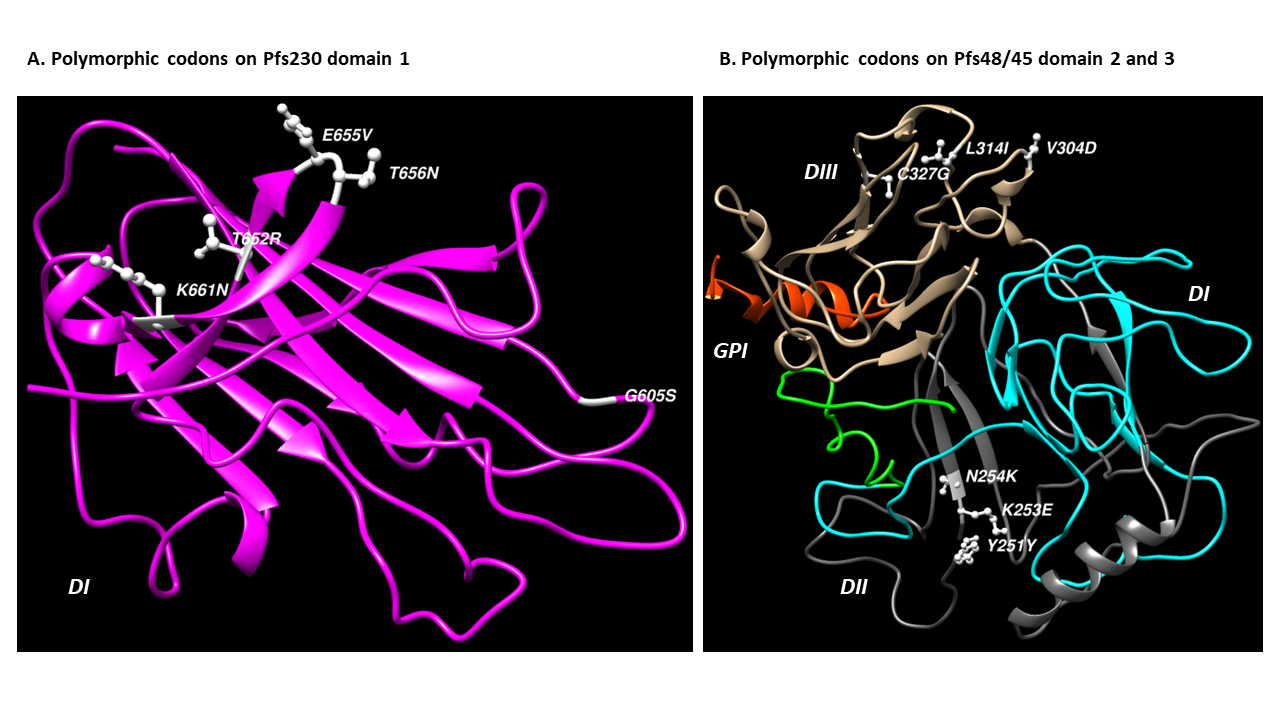

Supplement: Supplementary file 1 [file Image1.TIF]

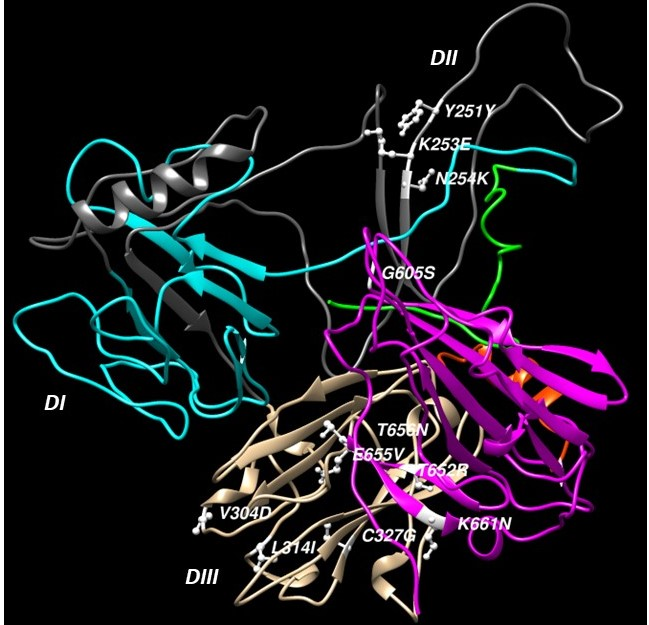

Supplement: Supplementary file 3 [file Image2.TIFF]
