# Supplementary material for: Rare Alleles and Signatures of Selection on the Immunodominant Domains of Pfs230 and Pfs48/45 in Malaria Parasites From Western Kenya
Source: Front Genet. 2022 May 17;13:867906. doi: 10.3389/fgene.2022.867906 (PMC9152164; doi:10.3389/fgene.2022.867906)
Supplement: Supplementary file 2 [file Table2.DOCX]

**SI Table**. **Haplotype frequencies and codon changes on Pfs230 domain 1 in Homa Bay, Kisumu and Kisii *Plasmodium falciparum* populations.** N: Sample size; Capital letters before numerical denotes amino acid change at respective codon; n: Proportion of sequences under respective haplotypes; LD: Linkage Disequilibrium; χ ^2^: Chi-square values; ^***B^: Highly significant by the Bonferroni procedure; ^ns^: Non-significant; D’: Parameter for estimating LD; r^2^: Parameter for estimating LD; Rm: Recombination events.

| **Homa Bay (N=82)** | | | | | | |
| --- | --- | --- | --- | --- | --- | --- |
| **Haplotype** | **Mutated codon** | **Haplotype**  **frequency n, (%)** | **LD**  **(Linked loci)** **χ 2** | **D’** | **r^2^** | **Rm** |
| Hap_1  (NF54) | 0 | 0.00 | (1813, 1955) 19.738***^B^  (1813,1983) 0.052^ns^  (1955,1983) 2.811^ns^ | 0.49  0.14  1.00 | 0.31  0.06×10^-2^  0.03 | (1813, 1955) |
| Hap_2 | G605S | 47 (57.32) |  |  |  |  |
| Hap_4 | G605S/K661N | 33 (40.24) |  |  |  |  |
| Hap_7 | G605S/T652R/K661N | 1(1.22) |  |  |  |  |
| Hap_8 | T652R/K661N | 1(1.22) |  |  |  |  |
| **Kisumu (N=39)** | | | | | | |
| Hap_1  (NF54) | 0 | 1(2.56) | (1616, 1813) 0.171^ns^  (1616, 1955) 0.054^ns^  (1616, 1967) 0.054^ns^  (1616, 1983) 1.905^ns^  (1813, 1955) 12.650*** ^B^  (1813, 1967) 0.083^ns^  (1813, 1983) 0.261^ns^  (1955, 1967) 0.026^ns^  (1955,1983) 1.134^ns^  (1967,1983) 1.134^ns^ | -1.00  -1.00  -1.00  -1.00  1.00  -1.00  -0.30  -1.00  1.00  1.00 | 0.38×10^-2^  0.14×10^-2^  0.14×10^-2^  0.05  0.32  0.21×10^-2^  0.66×10^-2^  0.07×10^-2^  0.03  0.03 | (1813, 1983) |
| Hap_2 | G605S | 17(43.59) |  |  |  |  |
| Hap_3 | I539T/G605S | 2(5.13) |  |  |  |  |
| Hap_4 | G605S/K661N | 17(43.59) |  |  |  |  |
| Hap_6 | G605S/T656N/K661N | 1(2.56) |  |  |  |  |
| Hap_8 | T652R/K661N | 1(2.56) |  |  |  |  |
| **Kisii (N=35)** | | | | | | |
| Hap_1  (NF54) | 0 | 0.00 | (1813, 1955) 0.061 ^ns^  (1813, 1964) 0.029 ^ns^  (1813, 1983) 1.150 ^ns^  (1955, 1964) 0.061 ^ns^  (1955, 1983) 1.895 ^ns^  (1964, 1983) 0.920 ^ns^ | -1.00  -1.00  1.00  -1.00  -1.00  -1.00 | 0.17×10^-2^  0.08×10^-2^  0.03  0.17×10^-2^  0.05  0.03 | - |
| Hap_2 | G605S | 16(45.71) |  |  |  |  |
| Hap_4 | G605S/K661N | 16(45.71) |  |  |  |  |
| Hap_5 | G605S/E655V/K661N | 1(2.86) |  |  |  |  |
| Hap_7 | G605S/T652R/K661N | 2(5.71) |  |  |  |  |

**S2 Table. Haplotype frequencies and defining codon changes on Pfs48/45 domain 3 across Homa Bay, Kisumu and Kisii *Plasmodium falciparum* population.** N: Sample size; Capital letters before numerical denotes amino acid change at respective codon; n: Proportion of sequences under respective haplotypes; LD: Linkage Disequilibrium; χ ^2^: Chi-square values; ^***B^: Highly significant by the Bonferroni procedure; ^ns^: Non-significant; D’: Parameter for estimating LD; r^2^: Parameter for estimating LD.

| **Homa Bay (N=36)** | | | | | |
| --- | --- | --- | --- | --- | --- |
| **Haplotype** | **Mutated codon** | **Haplotype frequency n, (%)** | **LD**  **(Linked loci) χ 2** | **D’** | **r^2^** |
| Hp_1(NF54) | 0 | 32 (88.89) | (911, 940) 0.091^ns^ | -1.00 | 0.25×10^-2^ |
| Hp_2 | V304D | 1 (2.78) |  |  |  |
| Hp_3 | L314I | 3 (8.33) |  |  |  |
| **Kisumu (N=44)** | | | | | |
| Hp_1(NF54) | 0 | 41 (93.18) | - | - | - |
| HP_3 | L314I | 3 (6.82) |  |  |  |
| **Kisii (N=38)** | | |  |  |  |
| Hp_1(NF54) | 0 | 28 (73.68) | (940, 979) 0.308 ^ns^ | -1.00 | 0.79×10^-2^ |
| Hp_3 | L314I | 9 (23.68) |  |  |  |
| Hp_4 | C327G | 1 (2.63) |  |  |  |
